# Identification of novel microRNA regulatory pathways associated with heterogeneous prostate cancer

**DOI:** 10.1186/1752-0509-7-S3-S6

**Published:** 2013-10-16

**Authors:** Yifei Tang, Wenying Yan, Jiajia Chen, Cheng Luo, Antti Kaipia, Bairong Shen

**Affiliations:** 1 Center for Systems Biology, Soochow University, Suzhou 215006, China; 2 Drug Discovery and Design Center, Shanghai Institute of Materia Medica, Chinese Academy of Sciences, Shanghai, China; 3 Department of Urology, Tampere University Hospital, 33521 Tamper, Finland

## Abstract

**Background:**

MicroRNAs (miRNAs) are potential regulators that contribute to the pathogenesis of cancer. Microarray technologies have been widely used to characterize aberrant miRNA expression patterns in cancer. Nevertheless, the miRNAs expression signatures identified for a same cancer differs among laboratories due to the cancer heterogeneity. In addition, how the deregulated miRNAs coordinately contribute to the tumourigenic process of prostate cancer remains elusive.

**Results:**

We evaluated five outlier detection algorithms that take into account the heterogeneity of cancer samples. ORT was selected as the best method and applied to four prostate cancer associated microRNA expression datasets. After microRNA target prediction and pathway enrichment mapping, 38 Gene Ontology terms, 16 KEGG pathways and 99 GeneGO pathways are found putative prostate cancer associated. Comparison with our previous studies, we identified two putative novel pathways important in prostate cancer. The two novel pathways are 1) ligand-independent activation of ESR1 and ESR2 and 2) membrane-bound ESR1: interaction with growth factors signalling.

**Conclusions:**

We proved that expression signatures of at the pathway level well address the cancer heterogeneity and are more consistent than at the miRNA/gene levels. Based on this observation, we identified putative novel microRNA regulatory pathways which will help us to elucidate the cooperative function of different microRNAs in prostate cancer.

## Background

MicroRNAs (miRNAs) are small non-coding RNAs of approximately 22-nucleotides. They play important roles in gene regulation at post-transcriptional level. They are able to repress the activity of complementary mRNAs by targeting the 3'-untranslated regions [1]. Release 19 of the miRBase database contains more than 2200 mature miRNA sequences for human [2]. Aberrant miRNA expression was shown related to the generation of cancer stem cells and the tumour genesis [3-5]. Microarray-based technologies have routinely been used for profiling molecular expression in cancer. Microarray allows simultaneous expression profiling of tens of thousands of genes in normal versus malignant cells. The growing number of microarray expression datasets has necessitated the integrative analysis approaches to identify significant molecular patterns across multiple datasets.

Many efforts have been made in search of common molecular signatures, however without obvious success. This is partly due to the highly heterogeneous nature of cancer. Tumour samples often comprise of subpopulations with different genomic alterations. However, the most popular outlier detection algorithm, t-test or its analogues, simply removes heterogeneity between subtypes, and fail to identify the subgroup-specific gene alterations [6-8]. Recently novel statistical methods were developed to identify patterns only existed in the subgroups of the studied samples [9-13].

In this study, we applied these outlier detection methods to analyze our collection of four miRNA expression microarray datasets to identify differentially expressed miRNAs (DE-miRNAs). The DE-miRNAs were then compared among the four data sets at both gene and gene set (i.e., the functional gene set or pathway) levels for comparison. By considering the cancer heterogeneity, we applied different statistical methods to identify the consistent prostate cancer (PCa) associated pathways that are coordinately targeted by miRNAs.

## Results

### Comparison of heterogeneous feature detection algorithms

Most of the previous expression data studies used fold-change, t-test and other statistics alike to detect cancer-related genes. Recently, it has been recognized that many oncogenes show altered expression in only a small proportion of cancer samples [11]. Such features will be removed when using t-test or t-test like methods because they average gene expression levels in all the studied samples. Tomlins et al. concluded that t-tests were not adequate for detecting heterogeneous patterns of oncogenes [14].

To address this complexity, a series of new heterogeneous detection algorithms have been proposed in recent years. Among these methods are Least Sum of Ordered Subset Squared (LSOSS) [10], Cancer Outlier Profile Analysis (COPA) [9], Maximum Ordered Subset T-statistics (MOST) [11], Outlier Robust T-statistics (ORT) [13], and Outlier Sum (OS) [12].

**Figure 1:**
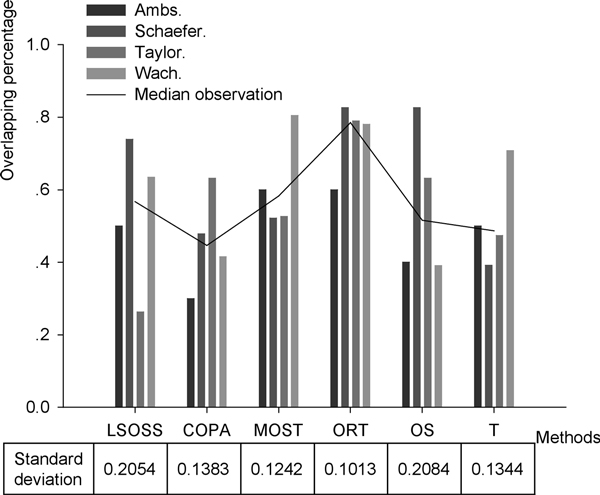
**Overlapping percentages of putative outliers (see text for definition)**. Outliers detected by at least three methods were considered to be putative ones.

The performance of the above algorithms and the traditional t-test were compared on the detection of the outliers in our collection of prostate cancer (PCa) associated microRNA expression data. The outliers here refer to the deferentially expressed microRNAs (DE-miRNAs). For all these methods applied to the different data sets with different numbers of samples, we set the quantile of outliers to 0.05 (5%). Those DE-miRNAs detected by at least three methods were considered to be putative PCa associated outliers, and then the percentages of the putative outliers in the original result of each method were calculated to determine the method's accuracy (see Figure 1). In most of the cases, these heterogeneity feature detection algorithms performed better than the traditional t-test. In most of this comparison, ORT performed better than the other methods. For these four studied datasets, ORT had the biggest median observation and smallest standard deviation. Therefore, we take the result by ORT for the downstream analyses.

### The outlier miRNA targets in prostate cancer

As miRNAs play a role in post-transcriptional regulation by targeting complementary mRNAs, we collection their putative targets and subsequently mapped these target genes to pathways or gene sets for enrichment analysis. Target genes were retrieved from both TargetScan database and our integrative prediction (see methods section for detail). Additional file 1 shows the target genes of the PCa associated DE-miRNAs. At last, 1236, 3566, 1520 and 4749 target genes of the DE-miRNAs of four different datasets were obtained respectively.

### The identification of the microRNA regulatory pathways in prostate cancer

**Figure 2:**
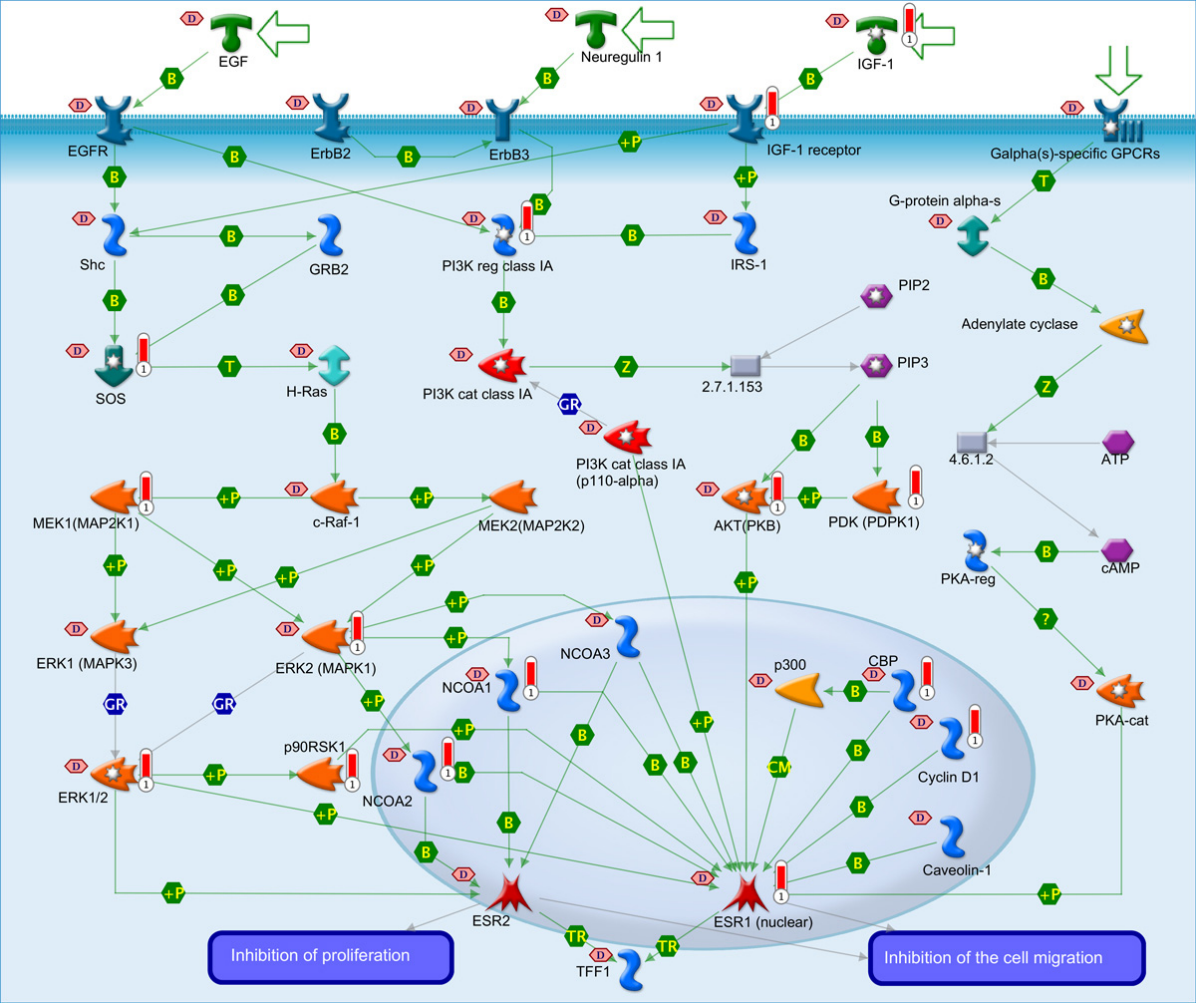
**The most significant GeneGO pathway map**. Development: Ligand-independent activation of ESR1 and ESR2. Additional file 2 shows the legend for this map. The target genes of the putative DE-miRNA are denoted by red bars. The light red hexagon labelled "D" denotes an association with prostate cancer.

The collection of the four different datasets are from different platforms, the overlapping of miRNA probes between these data are about 40~60% while the detected differently expressed miRNA profiles only have 3% overlapping [15]. We aim to identify the consistent pattern at high level. First, the target genes of DE-miRNAs found by at least 3 datasets were extracted, then mapped to function and pathway databases, e.g. GO [16], KEGG [17,18] and GeneGO (GeneGo, Inc), to identify PCa-associated functions and pathways. In this process, we identified 1221 target genes of the PCa associated DE-miRNAs, among which 253 were shared by all the four target gene datasets, and 968 overlapped in three of the four datasets. As shown in Figure 2, the ligand-independent activation of ESR1 and ESR2 is the most significant GeneGO pathway (See Additional file 2 for the notation of the symbols in this figure). In Figure 2, insulin-like growth factor-1 (IGF-1) encodes the protein involved in mediating growth and development. In this pathway, IGF-1 binds to IGF-1 receptor on the membrane and activates signal transduction through Shc, SOS, Mek1, and ERK2, finally mediating the production of ESR1 and ESR2. Genes involved in the signal transduction above are all target genes of highly expressed miRNAs in prostate cancer samples; therefore, the expression of ESR1 and ESR2 will be down-regulated which is in accordance with the previous report by Gamba and his co-authors [19].

**Figure 3:**
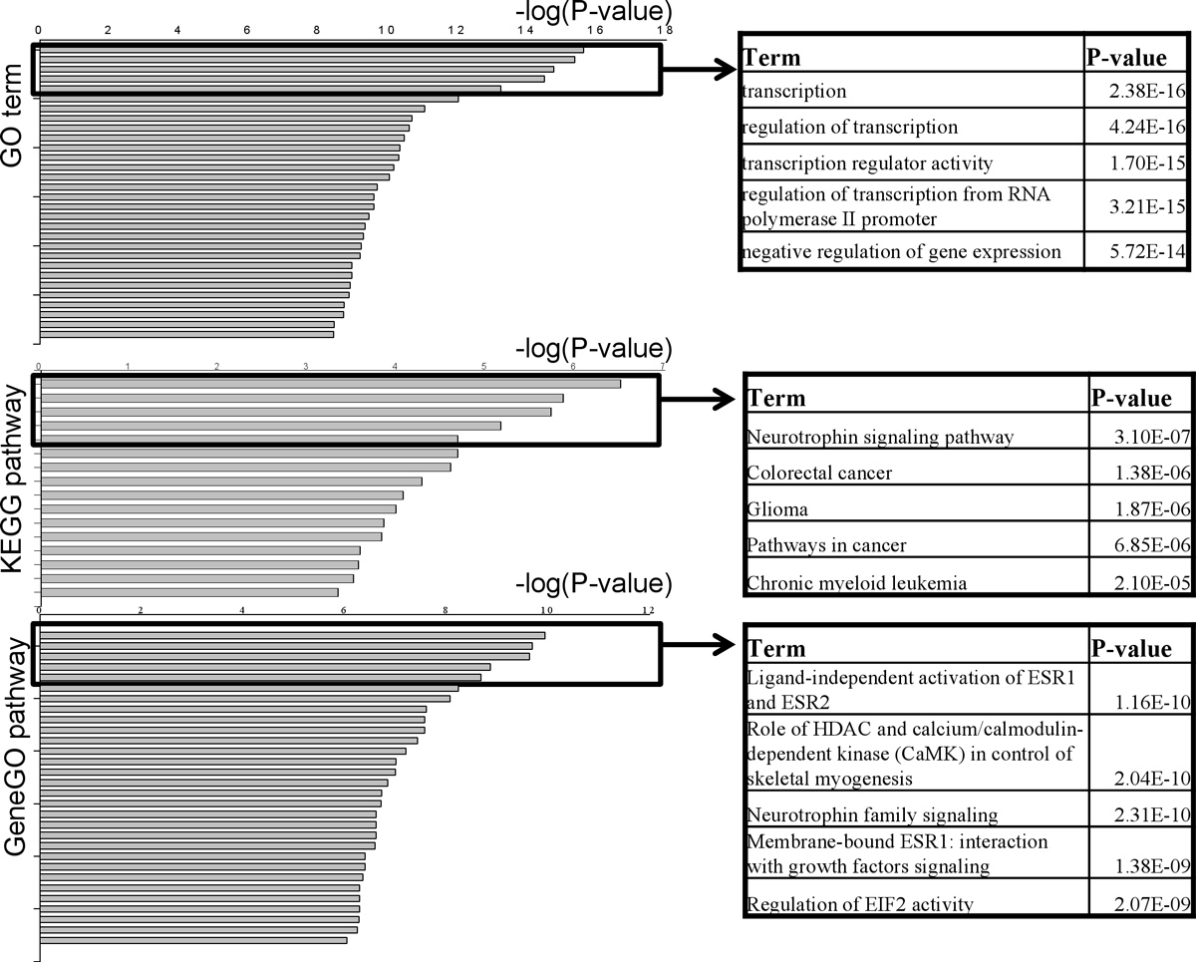
**Illustration of biological theme enrichment**. DE-miRNAs shared by at least three datasets were extracted to identify target genes; these genes were then mapped to databases to identify enriched GO terms (FDR < 0.001), KEGG pathways (p < 0.001), and GeneGO pathways (FDR < 0.001). Top GO terms, KEGG, and GeneGO pathways are shown. Terms shown in the box to the right of each bar plot are the most significant ones. Details are available in Additional files 3, 4, 5.

Figure 3 illustrates various biological themes enriched in the gene list. The left side of the figure is a bar plot of enriched GO terms, KEGG pathways, and GeneGO pathways against -log10 (p value); the top five terms of each biological theme were shown in the right. The details are also available in Additional files 3, 4, and 5. In these files, the pathway or GO terms were sorted by p value. Overall, we identified 38 GO terms (FDR < 0.001), 16 KEGG pathways (*p *< 0.001), and 99 GeneGO pathways (FDR < 0.001) that are enriched with target genes of the PCa associated DE-miRNAs.

### Analysis and validation of the putative microRNA regulatory pathways in prostate cancer

Among the 99 enriched GeneGO pathways, 67 (67.7%) pathways were also significantly enriched in our previous study in which we processed 10 mRNA microarray datasets [20]. In the set of top 15 GeneGO pathways in our previous work, 11 (73.3%) were also detected in the 99 pathways in this study (see Additional file 5).

To identify potential microRNA regulatory pathways in prostate cancer, the 15 most significantly enriched (i.e., with the lowest p value) pathways were chosen for the analysis. Of those, four had previously been reported to be related to prostate cancer in PubMed citations. We verified the other 11 pathways indirectly by analysis of the component genes in PubMed citations although the wet-lab experiments can direct validate them (Table 1). Among the top 15 pathways reported by both the previously and the present studies, 3 pathways are the same in both studies, 2 of the 3 pathways are novel ones *i.e*., 1) ligand-independent activation of ESR1 and ESR2, this is the most significant pathway we mentioned in the last section, and 2) membrane-bound ESR1: interaction with growth factors signalling.

**Table 1:** Top 15 enriched GeneGO pathways.

Category	Term	PubMed citation count*
Development	Ligand-independent activation of ESR1 and ESR2^§^	
Development	Role of HDAC and calcium / calmodulin-dependent kinase (CaMK) in control of skeletal myogenesis	
Development	Neurotrophin family signalling	
Development	Membrane-bound ESR1: interaction with growth factors signalling§	
Translation	Regulation of EIF2 activity	
Translation	Insulin regulation of translation	1
Development	IGF-1 receptor signalling	3
Transcription	Receptor-mediated HIF regulation	
Immune response	IL-15 signalling	
Development	PIP3 signalling in cardiac myocytes	
Signal transduction	Activin A signalling regulation	
Apoptosis and survival	BAD phosphorylation §	10
Neurophysiological process	NMDA-dependent postsynaptic long-term potentiation in CA1 hippocampal neurons	
G-protein signalling	Proinsulin C-peptide signalling	1
Development	Thrombopoietin-regulated cell processes	

*The citation count was calculated by searching the pathway names in title and abstract fields of PubMed.

^§^Pathways with this mark are also in the top15 pathways of our previous study [20].

The following two pathways are novel pathways identified by both the present and the previous studies. 1) Ligand-independent activation of ESR1 and ESR2; 2) Membrane-bound ESR1: interaction with growth factors signalling.

PubMed citation counts of corresponding genes in each potential pathway can be found in Additional file 6. According to PubMed citation results, the percentages of reported PCa related genes in each pathway range from 25.0% to 71.4%. These percentages will be changed with the PubMed update, since more researches were performed to investigate the caner hallmarks related pathways, some pathways may be overrepresented in the PubMed database while others may have less citations. The results of PubMed citations indirectly verified the link between the pathways and the prostate cancer, although experimental validation is needed for further confirmation.

## Discussion

In this study, we collected four prostate cancer miRNA microarray datasets. These datasets were processed with outlier detection statistical methods considering cancer heterogeneity. This is the first work to compare the performance of heterogeneity feature detection statistical methods with real miRNA datasets. The analysis indicates these novel algorithms generally perform better than the t-test. All the methods are important and they may show different performance for different data sets, we could select the best methods based on the consensus analysis.

Figure 3 illustrates the GO terms or pathways (both from KEGG and GeneGO) that are enriched with the overlapped target genes from the PCa DE-miRNAs of the four datasets. The top 5 enriched GO terms are all related to transcription and its regulation, which are in accordance with the observation of the abnormal gene expression in prostate tumours. Most of the identified significant KEGG or GeneGO pathways are important for cancer developing and usually involved in the gene expression or tumour metastasis. Neurotrophins exert their functions by engaging Trk tyrosine kinase receptors or p75 neurotrophin receptor (p75 NTR), a metastasis and tumour suppressor in prostate cancer [21,22]. ESR1 inhibits cell migration and the repression of ESR1 expression enhances cell migration and accelerates tumour formation and metastasis. All the evidence above corroborates our findings in the present study.

The comparison of the previous study [20] with the present one indicates the high consistency between the integrative analysis of the microRNA and the mRNA microarray expression datasets. We here identified 11 novel PCa associated pathways (see Table 1). Two novel pathways among the top 15 in both studies are identified. These overlapping pathways can be potential key pathways contributing to prostate carcinogenesis. Among the key genes in these two novel pathways, histone deacetylaces (HDACs) was reported abnormally expressed in prostate cancer [23]. Additionally, the IGF family is involved in the regulation of prostate growth and bone metastasis [24]. In prostate cancer cells, the IGF-1 receptor, a tyrosine kinase receptor related to tumour progression and metastasis, is highly expressed with MT1-MMP, a metalloproteinase involved in prostate cancer metastasis [25]. Abnormal HIF expression mediates vital processes such as cell survival, proliferation, and angiogenesis [26,27]. Activin A inhibits prostatic branching and growth [28] and enhances prostate cancer cell migration [29]. Additionally, IL15 activates neutrophils and dendritic cells and generates cytotoxic T lymphocytes against cancer cells [30], so the blocking of the IL15 signalling pathway weakens the immune system's ability to resist cancers. Additional file 6 shows the PubMed citation counts of corresponding genes of each potential pathway in prostate cancer. More wet-lab experiments are suggested to verify the functions of these pathways in prostate cancer.

## Conclusions

In this study, heterogeneity feature detection methods were evaluated and applied to the identification of the novel microRNA regulatory pathways in prostate cancer and 11 novel PCa associated pathways were identified. Comparing the present study on PCa microRNA expression data with our previous work on PCa gene expression data, we identified two important novel pathways among the top 15 of the two studies.

## Methods

### Data collection

**Figure 4:**
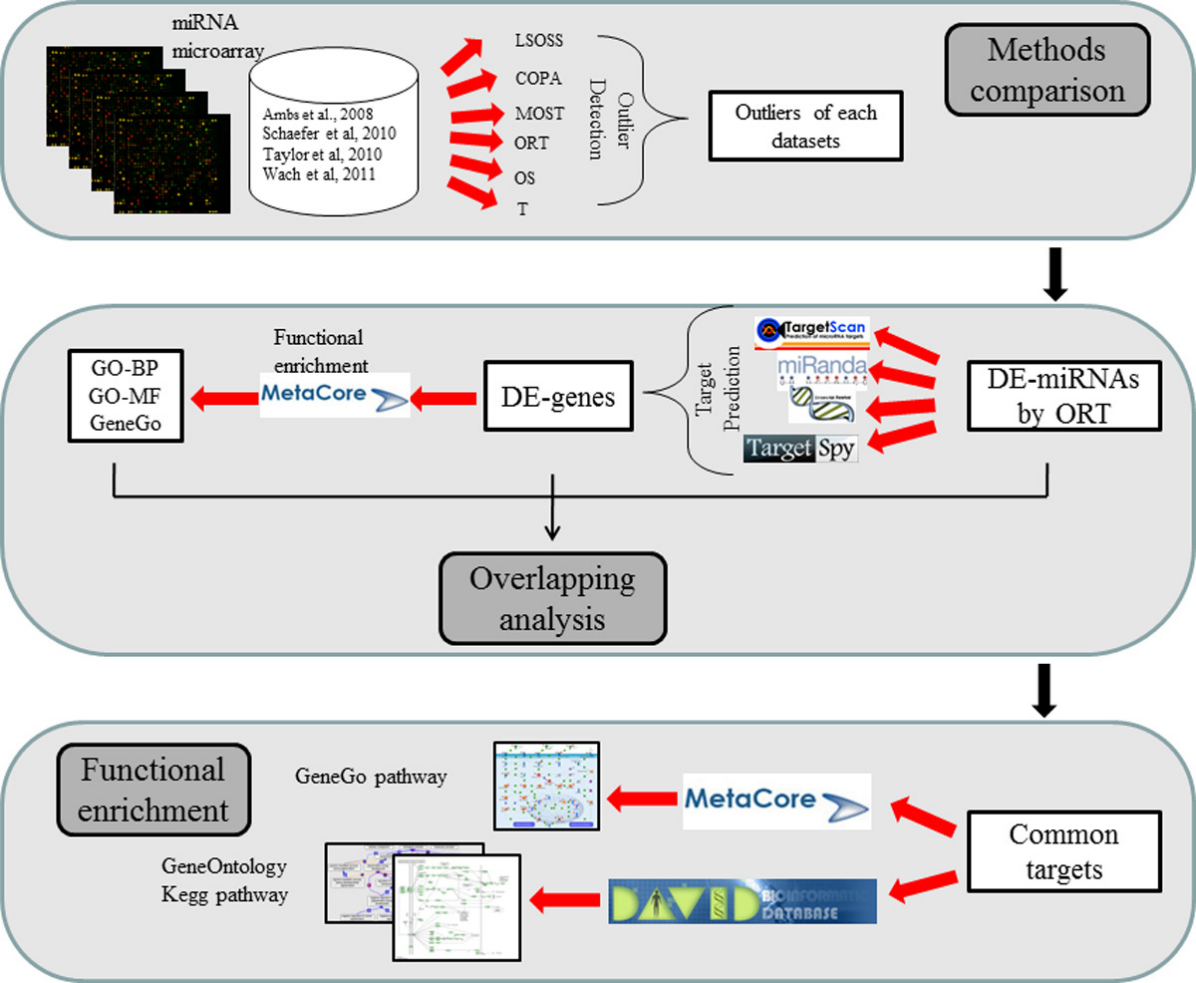
**The pipeline of the whole procedure used in this study**.

We retrieved four miRNA expression datasets from Gene Expression Omnibus (GEO, http://www.ncbi.nlm.nih.gov/geo/), which is a public functional genomics data repository supporting MIAME-compliant data submissions. The datasets were downloaded in single-matrix file format, and named according to the name of the first authors of the references (Table 2). The miRNA probes of these datasets were designed by using Sanger miRBase release 16.0. Because of the diverse platforms of the datasets, a local Blast search [31] was performed by mapping probe sequences to the miRNA precursors of miRBase (release 16.0 [2]) to identify the concordant miRNA names. Figure 4 displays the pipeline of the whole procedure used in this study.

**Table 2:** Prostate tissue datasets used in this study.

Dataset	**GEO accession NO**.	Platforms	Human miRNA probes	Number of samples		Statistics	**Ref**.
						
				Prostate normal tissue	Prostate cancer tissue		
Ambs	GSE8126	OSU-CCC hsa-miRNA-chip version 3	474	16	60	T-test	[35]
Schaefer	GSE14857	Agilent-016436	407	12	12	T-test	[36]
Taylor	GSE21036	Agilent-019118	373	28	113	Mixture model	[37]
Wach	GSE23022	Affymetrix miRNA Array	847	20	20	ANOVA	[38]

### Comparison of detection algorithms and detect the differentially expressed miRNAs

In this study, outliers of microRNA expression in PCa microarray datasets were detected by using six statistical methods: LSOSS, COPA, MOST, ORT, OS and t-test. All these methods were implemented in R packages written by Wang [10] and Lian [11]. The quantile of outlier extraction for all the methods was set to 0.05 (5%) by default.

We compared the performance of the six methods in obtaining the PCa associated DE-miRNAs. We considered the DE-miRNAs detected by at least three methods to be putative outliers. The percentage of these putative outliers in the original result of each method was calculated to measure the method's accuracy. We selected ORT to be the best method for these PCa microRNA expression datasets considering the consensus analysis results.

### Reliable prediction of targets for PCa DE-miRNAs

Targets of DE-miRNAs were retrieved from TargetScan database by a series of in-house Perl scripts. For those miRNAs unavailable in the TargetScan database, the putative targets were manually predicted by performing a genome-wide, sequence-based bioinformatics procedure with three of the most popular tools, i.e., miRanda [32], RNAhybrid [33], and TargetSpy [34]. Only the overlapped targets of the prediction were kept as reliable result.

### PubMed Search and the citation counts

PubMed citation count was calculated by searching PubMed in the fields of title and abstract, such as for the "ligand-independent activation of ESR1" pathway, we use "ligand-independent activation of ESR1 [tiab] AND prostate cancer [tiab]" as the search term, and the search term "SP1 [tiab] AND prostate cancer [tiab]" was applied to the search of the link between SP1 gene and prostate cancer. This citation counts may change with the update of PubMed.

### GO and pathway enrichment analysis

To study the function of the PCa DE-miRNAs, we mapped their target genes to GO, KEGG and GeneGO databases. To decrease the number of the false positives pathways, we first identified target genes shared by at least three PCa DE-miRNAs datasets, which were then mapped to GO, KEGG pathway database by DAVID, and GeneGO pathway database by MetaCore (Gene, Inc.). Both DAVID and MetaCore use hypergeometric distribution to calculate the significance level (i.e. the *p *value) for each pathway and adjust it using the FDR value as the threshold. In MetaCore databases, p value means the probability of a random intersection of two gene sets, with low p values indicating a high potential of non-randomness of the finding.

## Competing interests

The authors declare that they have no competing interests.

## Authors' contributions

YT, JC and WY carried out the calculation and analysis, AK and CL participated in the discussion of the project and drafted the manuscript; BS conceived the idea and revised the manuscript. All authors read and approved the final manuscript.

## Supplementary Material

Additional file 1 **Entrez IDs and Official Symbols of targeting genes for DE-miRNAs of each datasets**. Click here for file

Additional file 2 The notations of all the symbols in Figure 2. Click here for file

Additional file 3 **Enriched GO terms with P-value and FDR**. Gene list was generated by extraction of target genes from at least 3 datasets. (FDR < 0.001) Click here for file

Additional file 4 **Enriched KEGG pathways with P-value**. Gene list was generated by extraction of targeting genes from at least 3 datasets. (P-value < 0.001) Click here for file

Additional file 5 **Enriched GeneGO pathways with P-value**. Gene list was generated by extraction of targeting genes from at least 3 datasets. (FDR<0.001) Click here for file

Additional file 6 **Citation counts of corresponding genes of each potential GeneGO pathway**. Citation count was calculated by searching PubMed in the fields of title and abstract, this may change with the update of PubMed. Click here for file

## Declarations

Publication of this article was funded by the National Natural Science Foundation of China grants (91230117, 31170795).

This article has been published as part of *BMC Systems Biology *Volume 7 Supplement 3, 2013: Twelfth International Conference on Bioinformatics (InCoB2013): Systems Biology. The full contents of the supplement are available online at http://www.biomedcentral.com/bmcsystbiol/supplements/7/S3.
