# Supplementary figures and images for: Identification of novel microRNA regulatory pathways associated with heterogeneous prostate cancer
Source: BMC Syst Biol. 2013 Oct 16;7(Suppl 3):S6. doi: 10.1186/1752-0509-7-S3-S6 (PMC3852103; doi:10.1186/1752-0509-7-S3-S6)

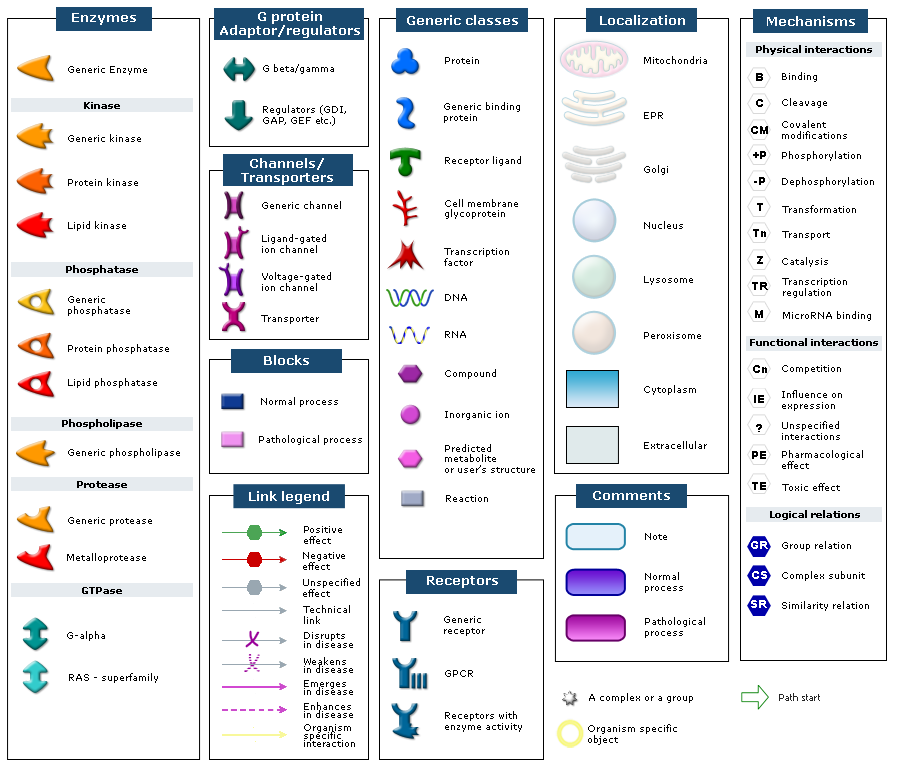

Supplement: Additional file 2 — The notations of all the symbols in Figure 2. [file 1752-0509-7-S3-S6-S2.tif]
